# PHYLIS: A Low-Cost Portable Visible Range Spectrometer for Soil and Plants

**DOI:** 10.3390/s17010099

**Published:** 2017-01-07

**Authors:** Matt J. Aitkenhead, Graham J. Gaskin, Noemie Lafouge, Cathy Hawes

**Affiliations:** 1 The James Hutton Institute, Invergowrie, Dundee DD2 5DA, UK; graham.gaskin@hutton.ac.uk (G.J.G.); cathy.hawes@hutton.ac.uk (C.H.); 2 Ecole d’Ingénieurs de Purpan, 75 Voie du Toec, 31076 Toulouse, France; noemie.lafouge@etudiants.purpan.fr

**Keywords:** spectroscopy, soil, agriculture, crop assessment, precision agriculture

## Abstract

Monitoring soil and crop condition is vital for the sustainable management of agricultural systems. Often, land management decision-making requires rapid assessment of conditions, which is difficult if samples need to be taken and sent elsewhere for analysis. In recent years, advances in field-based spectroscopy have led to improvements in real-time monitoring; however, the cost of equipment and user training still makes it inaccessible for most land managers. At the James Hutton Institute, we have developed a low-cost visible wavelength hyperspectral device intended to provide rapid field-based assessment of soil and plant conditions. This device has been tested at the Institute’s research farm at Balruddery, linking field observations with existing sample analysis and crop type information. We show that it is possible to rapidly and easily acquire spectral information that enables site characteristics to be estimated. Improvements to the sensor and its potential uses are discussed.

## 1. Introduction

Assessment of crop condition and soil characteristics in agriculture is vital for maximising yields. Often, it would benefit growers to receive the results of an assessment as quickly as possible, as the crop condition and soil nutrient levels that drive management decisions can change rapidly. Currently, samples are sent off to a laboratory for analysis, which adds time and cost to the process. Any method that can allow rapid in-field assessment of soils and crops, or the rapid capture of data to allow office-based automated assessment will improve the ability of growers to make timely and informed day-to-day management decisions.

Field spectroscopy has potential in this area, as an approach that may allow high-quality data relating to soil and crop characteristics to be captured rapidly and non-invasively. Ref. [1] discussed the potential for hyperspectral scene analysis in agriculture, particularly for the detection of pests and diseases before they become visible to the human eye. In a review of infrared spectroscopy for in-field plant characterisation, Ref. [2] highlighted the potential of this particular wavelength range for compositional analysis and phenotyping of plants, while Ref. [3] demonstrated that mid-infrared spectroscopy could be used for assessment of soil properties following land use change. Ref. [4] identified the benefits of soil spectroscopy, including speed, cost-effectiveness and the fact that it can be used non-destructively. They also reviewed specific priority areas for improvement of the field, including the development of spectral libraries and standards.

Ref. [5] highlighted the potential for IR (Infrared) spectroscopy across agricultural practices in developing countries, with many of their points regarding diagnostics and support for land management decision support being valid for agriculture in developed countries as well. They also identified capacity-building that would be required in terms of centres of excellence, equipment design and the development of decision support systems. This work and others (e.g., [6]) highlight existing constraints on the use of hyperspectral equipment and developments that need to be made. One of the main areas where improvement is required is portability, although recent development of low-weight and low-cost hyperspectral systems for field spectroscopy is leading to devices light enough that they can be attached to small Unmanned Aerial Vehicles (UAVs) (e.g., [7]).

A number of different crop characteristics have been identified as amenable to spectroscopic evaluation. Identification and phenotyping using NIR (Near Infrared) spectroscopy have been found to discriminate dry leaves of different medicinal plant species [8]. Ref. [9] demonstrated a non-invasive portable spectrophotometer for in-field plant phenotyping, using data mining approaches for calibration development in the wavelength range 1600–2400 nm.

Related to plant identification, weed/crop discrimination is another potentially useful function. Ref. [10] used Vis-NIR (visible and near-infrared wavelength ranges) for discriminating between crop (tomato) and weed species. They found that NIR wavelengths gave good discrimination, as did hyperspectral visible-only data, but that broadband colour-only models were not as effective. Ref. [11] also explored the use of NIR spectroscopy for automated weed detection, with a view to targeted herbicide application. They found that the crop soybean could be distinguished from two weed species.

Disease detection and crop chemical composition are high on the list of in-field assessment goals. Ref. [12] demonstrated the effectiveness of NIR for assessment of seed quality for a number of grain crops. NIR spectroscopy of grains can be used to detect specific diseases [13] and estimate the protein content of wheat kernels [14].

Ref. [15] compared red-edge inflection point and chlorophyll concentration in canola and found a useful level of estimation accuracy. Ref. [16] used NIR to estimate the nutrient content of plant leaves and found varying degrees of accuracy: N could be estimated well; P, Fe and Mn were estimated moderately well; and K, Zn and Cu were poorly estimated.

In addition to nutrient content, crop quality is an important factor. Estimation of important taste- and storage-related mango fruit properties can be carried out using NIR in the wavelength range 1200–2200 nm [17]. This work also highlighted the importance of appropriate preprocessing of the spectral data to optimise property estimation accuracy. Ref. [18] showed that NIR could be used for grading the quality of cotton leaves automatically.

Nitrogen status is an important indicator of crop health, and when nitrogen supply problems develop, they need to be addressed rapidly. Estimation of N uptake in cereal crops can also provide a source of information for calculating variable fertiliser application rates within fields. Refs. [19,20] used NIR to estimate total N in above-ground biomass of cereal crops and showed that, while within-field spectroscopy calibrations produced good results, more general model calibrations across multiple fields or at larger spatial scales were problematic.

Ref. [21] identified the wavelength ranges useful for estimating nitrogen status in switchgrass and sorghum using a handheld spectrophotometer. They found that specific wavelength ranges in the visible and red-edge could be used for this, and argued further that spectroscopy was a viable tool in evaluating the biophysical and biochemical characteristics of energy crops.

In addition to the crops themselves, assessment of soil composition provides important information for management decision-making. Ref. [22] showed that different wavelength ranges can contribute information on soil total carbon estimates, and Ref. [23] demonstrated that visible wavelength spectroscopy (350–700 nm) could be used to estimate N, P and K in paddy soils, although they also showed that NIR was more effective for estimating N and K. This differs from the work of [16] mentioned above, which found that K was poorly estimated in plant leaves using NIR. This difference in calibration performance between plant and soil for certain characteristics is difficult to explain without a detailed comparison of the two approaches used, and highlights the fact that variation in materials and methodologies can make large differences in estimation accuracy.

Ref. [24] demonstrated a system for real-time estimation of soil properties with Vis-NIR and found a range of calibration accuracy from moderate to good. Ref. [25] evaluated the visible (350–700 nm) and NIR (700–2500 nm) wavelength ranges for estimation of soil properties in paddy soils. They found that, for most properties, NIR produced better estimation accuracy, but visible wavelengths produced better estimation for soil electrical conductivity and available P.

Ref. [26] compared Vis-NIR and Fourier Transform NIR (FT-NIR) spectrometry for estimation of a number of soil attributes including pH, N, P, K and organic matter content. They showed that pH and organic matter could be estimated effectively using Vis-NIR, but not N, P or K, and also found that the preprocessing of the data made a strong difference in the effectiveness of the system. Ref. [27] described a tractor-driven system for real-time acquisition of Vis-NIR soil spectroscopy. They described the design and operation of the system, and demonstrated an error rate of 1.22 g·kg^−1^ of soil organic carbon estimation.

Physical attributes of soil can be rapidly assessed using mid-infrared spectroscopy (MIRS), as demonstrated by [28] who applied Partial Least Square (PLS) regression after first-order derivation and smoothing. The attributes assessed included liquid limit, air-dried moisture content and cation exchange capacity, all of which are also useful for agricultural purposes. Ref. [29] also demonstrated Vis-NIR based approaches for soil organic matter content estimation with PLS.

As mentioned above, model calibration is an important factor in the performance of a hyperspectral crop/soil sensing system. Many different approaches exist, with the two most common possibly being PLS and neural networks. Ref. [30] used Vis-NIR and neural networks to develop a calibration capable of discriminating between soybean plants and weeds, while identification of nitrogen deficiency using a combination of NIR and neural networks can be carried out successfully [31].

Another major factor affecting model accuracy is the preprocessing of the spectra. Ref. [32] reviewed the use of spectroscopy for assessment of soil fertility indicators, and found that the use of appropriate preprocessing approaches was linked to calibration performance. They also showed that neural networks are a useful approach in this area. Ref. [33] carried out a comparison of different preprocessing and calibration approaches for estimating soil carbon using NIR, and found that while preprocessing did not cause an improvement, the integration of topographic parameters did increase accuracy. However, Ref. [34] evaluated a marketed Vis-NIR spectrophotometer in a series of comparisons for estimating chlorophyll content in soybean. They showed that the device could produce useful results, and that the accuracy of these results depended on how the spectra were preprocessed prior to model calibration. Any new method or technology should ensure that performance is optimised through appropriate calibration approach selection. Often, this is a process of trial and error, as it is practically impossible to determine beforehand which calibration or preprocessing approach will work best for a specific sensor/application combination.

In-field sensing also comes with the issue of sample lighting, as without additional equipment the user has to rely on sunlight for illumination. Ref. [35] used two wavelengths (610 and 1220 nm) to estimate nitrogen and chlorophyll content in crops, and also included a system for eliminating the effects of variable solar radiation. This can be an issue when carrying out reflectance spectroscopy in the field and using natural light as the source of illumination. An example of work on eliminating unwanted effects from natural light for visible-wavelength monitoring in the field is given in [36].

Much of the work referenced above used NIR or MIR, sometimes in combination with visible-range wavelengths. Sensors in the near- and mid-infrared are more expensive than visible-range sensors, implying greater equipment costs. While we accept that these wavelength ranges provide important information related to plant and soil properties, our intention here is to explore the potential of a device that operates only in the visible range. The rationale for this is to determine whether the resulting device, with appropriate data preprocessing, can still provide useful information while doing so cheaply and rapidly and with a minimum of support equipment (i.e., handheld and with no artificial lighting). Existing examples of research using visible light alone (e.g., [37,38]) indicate that, certainly for soil, estimates for certain variables could be achieved and that colour information captured using digital cameras can be of sufficiently good quality to achieve this.

## 2. Materials and Methods

### 2.1. PHYLIS

**Figure 1:**
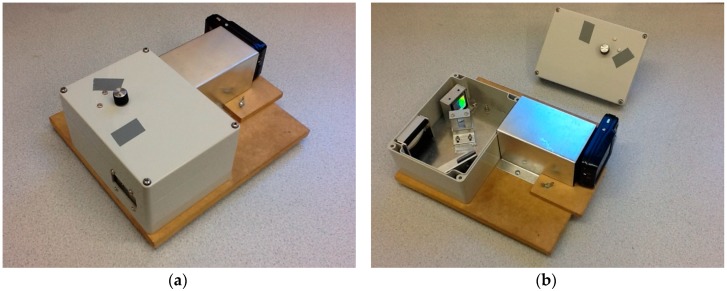
Photographs of the prototype sensor PHYLIS (Portable Hyperspectral Low-cost Imaging System): (**a**) PHYLIS with the optical equipment enclosed, as it is used in the field; (**b**) PHYLIS with the lid removed, showing the optical components.

The hyperspectral imaging system used in this work is a device developed and built at the James Hutton Institute. We have named it PHYLIS (Portable Hyperspectral Low-cost Imaging System). PHYLIS is a prototype device and the first version was constructed almost entirely from spare parts available in the Institute workshops. Later versions have been adapted slightly and the overall design improved, but the fundamental operation is the same. Figure 1 shows the components of PHYLIS with the light cover removed.

Light enters the device through a cylindrical lens and is collimated before falling onto a diffraction grating. This diffracts the light onto a relatively simple design of mirrors and lenses and produces a spectrum of visible wavelengths that is captured by a small, cheap digital camera (Vivitar (Edison, NJ, USA) Vivicam F128, 3 megapixels, approximately 20 Euros,). A control wheel mounted on the top of the box adjusts a slit aperture for light entering the device, controlling the level of light intensity that is taken in the photograph without altering the shape of the spectrum.

**Figure 2:**
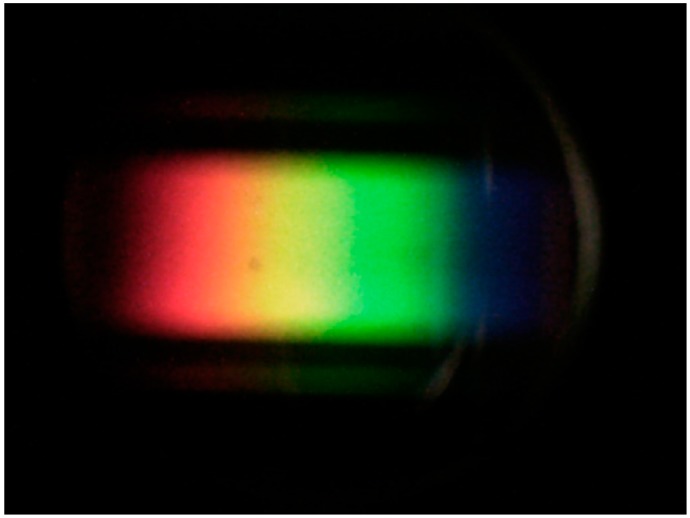
An example of a spectrum captured using PHYLIS, as taken by the camera attached to the device.

The captured image contains the spectra from the field of view of the device, which is approximately 2° vertically and 0.5° horizontally. The whole device is mounted on a wooden board that can be fixed to a camera tripod, although, throughout this work, all spectra were captured when the PHYLIS was handheld. Figure 2 gives an example of the images captured by the digital camera.

**Figure 3:**
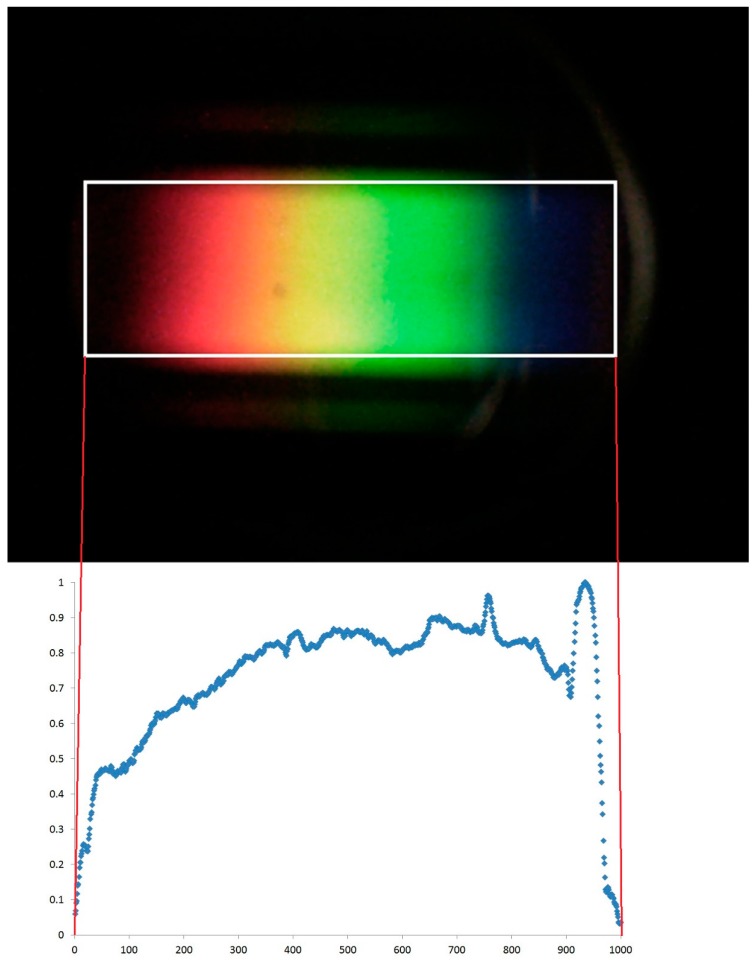
A schematic demonstrating the conversion of the photograph captured by PHYLIS into a spectrum.

The position of each column is therefore calculated along an arbitrarily-chosen scale of 1000, with 1 being the left-hand side of the bounding box and 1000 being the right-hand side. In practice, the number of columns in the bounding box is almost always between 700 and 800 pixels, resulting in small gaps, one character wide, in the data. These gaps are filled by taking the mean intensity values of neighbouring values and inserting them into the gaps. See Figure 3 for a schematic explaining this process.

After the images have been captured, they are downloaded directly from the camera using a USB cable and processed using bespoke software developed using Microsoft Visual Studio 2010 (Microsoft, Redmond, WA, USA). This can be done in the field using a laptop, although it is easier in an office environment given the current “prototype” nature of the system. The software extracts the pixel intensity values (RGB—red, green, blue values in the range 0–255). It uses these intensity values to identify the circular area enclosing the spectrum (see Figure 2). Due to the design of PHYLIS, the spectra are always in precisely the same region in this circular area, but the circle itself can be offset slightly and vary slightly in size within the image. This is because the camera itself occasionally needs to be moved to replace batteries, and therefore is not always replaced in exactly the same position.

Following identification of the region of interest within the image, a bounding box is placed around the region and the mean total pixel intensity (sum of RGB values) determined for each column of pixels within this box. It is assumed that this mean intensity represents the reflectance at individual wavelengths. The bounding box length corresponds to the same spectral range in each image, but does not have the same number of columns every time. This is for the same reason as the variable position and size of the circular area in each photograph. Based on evaluation using light sources with known spectral characteristics, we have calculated that the lower and upper wavelength limits of the bounding box are 390 and 700 nm, respectively.

### 2.2. Vegetable Garden Samples

In late June 2016, samples of different fruit and vegetable plants were imaged under natural lighting conditions at a community garden plot in Aberdeen. This imaging was carried out in one two-hour period, during which light levels were consistently high and mildly overcast. Leaves of four examples of twelve crops were imaged using PHYLIS, with two samples per plant taken from different leaves (and, in most cases, different plants). Each crop type was noted and included the following:

- Strawberry
- Rocket (rucola)
- Pea
- Onion
- Garlic
- Broad bean (fava bean)
- Broccoli
- Early potato
- Maincrop potato
- Tomato
- Courgette
- Chilli

Analysis of the spectra from the vegetable plot was made to determine if there were obvious differences between spectra due to crop type, and whether the two spectra from each crop were more similar than spectra from different crops. Two spectra from each of twelve crops is an insufficient data set to carry out full statistical analysis, and so comparison was made using dendrograms developed with distance measures between crop spectra. An investigation of different preprocessing methods was also included, with each set of preprocessed spectra used to produce dendrograms for comparison. The following commonly-used spectral preprocessing methods were applied: (1) none; (2) first-order derivative; (3) second-order derivative; and (4) Savitsky–Golay first-order smoothing.

Each of the above preprocessing options was carried out alone, and also followed by moving window subtraction (moving window radius of 20). All preprocessing steps were followed by normalisation of the resulting values to an absolute maximum of 1.

### 2.3. Light Level Analysis

At the time of spectral sampling in the vegetable plot, leaf samples from six crops were also taken (broad bean, pea, early potato, maincrop potato, strawberry and tomato). These were imaged using PHYLIS under controlled lighting conditions (tungsten bulb), with the lighting control wheel on PHYLIS set to six different light levels, with the same approximately equally-spaced levels used for each crop set between “maximum light input” and “zero light input”. Spectra were captured at each light level for each crop, and the same statistical evaluations carried out as listed above, using the preprocessing option that produced the best results for the spectra captured in-field.

### 2.4. Agricultural Crop Sampling

The James Hutton Institute’s Centre for Sustainable Cropping (CSC) at Balruddery was used to capture a set of spectra under natural light conditions. The CSC is a long-term experimental platform comprising a 42 ha block of six fields, established in 2009 to integrate cross-disciplinary research on sustainability in arable ecosystems. The effects of the sustainable versus conventional cropping systems are tested using a split-field design over a six course rotation (potato, winter wheat, field beans, spring barley, winter oilseed rape and winter barley). The six fields are divided into two, separated by a 6 m beetle bank buffer strip, and the cropping system treatments are randomly allocated to each half.

Conventional management follows standard commercial practice for each crop for the region. Sustainable management includes a range of practices aiming to maintain reasonable yields with less agrochemical inputs, resulting in enhanced biodiversity and reduced environmental pollution. These include minimum tillage to improve soil physical structure and reduce disturbance, compost addition and straw incorporation to enhance soil carbon content, reduce mineral fertiliser to be replaced with renewable sources of plant nutrients and atmospheric nitrogen fixation by legumes, cover crops to retain nutrients and reduce erosion, and lower rates of pest control products, compensated for by Integrated Pest Management strategies.

Within each treatment, five different cultivars of each crop type are sown in 18 m wide strips along the length of the field to test for a variety of specific responses to treatment. Within each strip, five permanent GPS sample locations are used to monitor a suite of system indicators throughout each growing season [39]. For this study, four of the six crops available (faba beans, potatoes, spring barley and winter wheat) were imaged using PHYLIS during July and August 2016. Two spectra were captured at three points in two of the variety strips for each half field, where soil samples had been collected in March of the same year. This provided a total of 96 spectra, with variation in crop type, cropping system (sustainable/conventional) and crop variety.

The spectra were preprocessed using the methods described above and a neural network (NN) model trained with the resulting data. Neural networks can be considered either as analogous to biological learning systems or as arrays of multiple parallel simultaneous relationships between model input and output variables; essentially, they are networks of connections between inputs, hidden layers of nodes and outputs with adjustable weightings. These weightings are altered in response to the disagreement between the model output and the “target” output over several thousand iterations of exposure to the model training data, with the weight adjustment being directed by one of many possible methods.

In this case, the NN was trained to discriminate between crops and cropping systems, using the commonly-applied backpropagation training algorithm. The model therefore had six outputs, one for each crop type and two for the different cropping systems (sustainable and conventional). The NN had two hidden layers each of 20 nodes and a training rate of 0.05. The training was carried out using 10-fold cross-validation, with the data split randomly between ten approximately equally-sized subsets. Each “fold” involved training one neural network model with nine of the ten subsets, and using the tenth for validation. Each subset was used as validation for one of the ten models. In addition, we ensured that the two spectra from each sample location were kept together in the same data subset. This was done to avoid artificially high validation scores that could be achieved by testing a model using a data point that was “twinned” with another in the training set.

Evaluation of the performance of the models was carried out using a confusion matrix, giving the number of times each crop type was identified as each of the possible types. Identification as one crop type or another was achieved using the “winner-takes-all” method of identifying the NN output node with the greatest output value, with each output node associated with one crop type.

### 2.5. Linkage to Agricultural Soil

Soil samples from each of the GPS locations at the CSC were analysed for eighteen elemental and chemical properties (see Table 3) to test for an association with crop spectral characteristics. Significant correlations between soil chemistry and crop spectral characteristics could provide a useful tool for identifying plant nutrient deficiencies in soils without the need for expensive soil testing.

A neural network model was applied similarly to that for the crop identification above. However, in this case, the outputs were not crop type, but the eighteen soil properties. Inputs to the model came from the preprocessed spectral data as before, with separate models for each preprocessing. There was insufficient data to train separately on each crop type, and so each NN model was “blind” to the crop that is used in each case. The 10-fold cross-validation approach was used as above, with identical parameterisation of the NN models.

Statistical evaluation of the results was carried out by determining the r-squared value of the linear regression between target and actual output values for each output variable, and the RMSE (Root Mean Squared Error) of the target vs. actual output values.

The two different data interpretation approaches used (dendrograms and neural network models) were selected based on the number of data points available in each case. It was felt that applying the neural network model to discrimination of crop types from the vegetable plot data would not have been scientifically robust, and so a dendrogram approach was applied. For the estimation of soil properties, sufficient data points were available to apply the 10-fold cross-validation approach to the estimation of continuous variables.

## 3. Results

### 3.1. Vegetable Garden Samples

**Figure 4:**
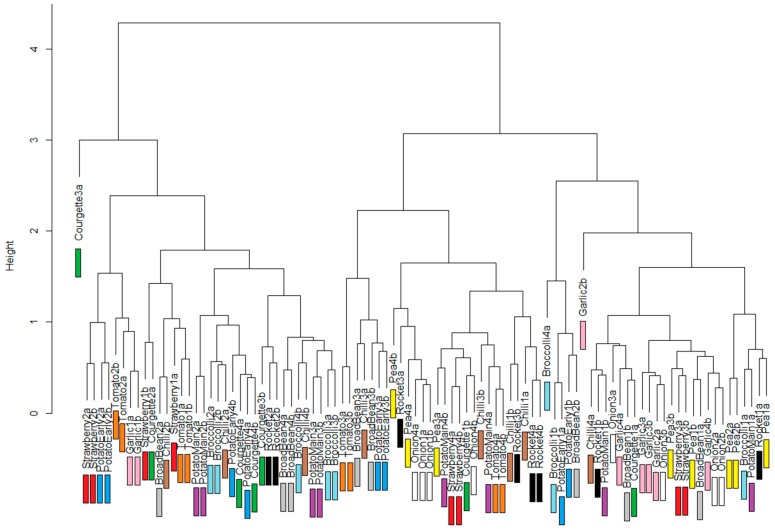
Dendrogram produced for the “no preprocessing”option on vegetable plot spectra.

Figure 4 shows the dendrogram produced from the summed Euclidean distance measures between individual spectra when no preprocessing was used. This was found to be the most meaningful option, as every other preprocessing option explored produced dendrograms that showed less clustering between spectra from the same crops, and a more complex dendrogram structure. Crop-specific colours have been added to Figure 4 to aid in identifying crop types.

The spectra captured for each crop tend to cluster together, meaning that they are more similar to each other than to other crop types (Figure 4). The clustering in Figure 4 also shows that certain crops are more spectrally similar to one another than they are to others. The most obvious example is the visible clustering of onions, peas and garlic. The use of Euclidean distance measures between individual spectra is only one way of measuring difference and is somewhat of a “blunt instrument” in discriminating between crops (as would be other distance measures such as Jaccard or Ecological, in summarising differences from 1000 pairs of values in two spectra down to a single metric). However, it does provide evidence that within-crop spectral variation is less than between-crop variation for a large number of fruit and vegetable crop plants.

### 3.2. Variable Light Levels

**Figure 5:**
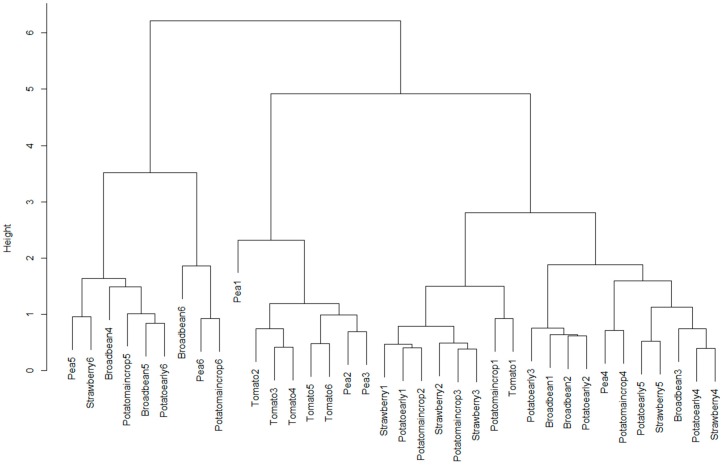
Dendrogram produced for the “no preprocessing” option and variable light levels on vegetable plot samples.

Figure 5 shows a dendrogram for spectra captured under different illumination levels for broad bean, pea, early potato, maincrop potato, strawberry and tomato leaves. This shows that while the clustering is not perfect for each crop, spectral variation for individual crops is less than that between crops. Broad bean spectra are relatively scattered across the dendrogram but are closely “related” in two different locations. Pea is poorly related here with only one “close” pairing. Early potato and maincrop potato are not individually discriminated; however, taken as one group of spectra, there are several examples in close proximity—admittedly, there are also several spread out as well. Three of the strawberry spectra are clustered closely (these are the brightest three) while the three “darker” strawberry spectra are scattered. Tomato spectra are well clustered, with five out of the six being closely clustered. This provides evidence that even at different light levels, some crops remain spectrally similar. However, it also shows that the identification of some crops may be sensitive to lighting conditions.

Evaluation of the dendrograms produced using different preprocessing options for the variable light level spectra gave a similar result to that for the in-field vegetable garden spectra: simple normalisation with no additional preprocessing gave the most meaningful spectral separability. The dendrograms for other preprocessing options had much more ‘random’ structures with no obvious clustering of crop spectra.

### 3.3. Agricultural Crop Samples

The results of the neural network model trained to discriminate between four crop types and two cropping systems are shown in Table 1 and Table 2. These tables give the confusion matrices for the categories and show that (a) beans, potatoes and grain crops are distinguishable as classes, but the grain crop classes are not (total accuracy of 67% for a four-class system, Kohen’s kappa of 0.56), and that (b) the two cropping systems are partially distinguishable using the crop spectra, but not very strongly distinguishable (total accuracy of 60% for a two-class system, Kohen’s kappa of 0.21).

### 3.4. Agricultural Soil Estimation

Table 3 gives the evaluation of the calibration used to estimate soil properties from crop spectra. The model performance varied greatly between properties, with some (e.g., NO_3_, available N, Mn) being well estimated with an r-squared value of greater than 0.5, and several others (e.g., pH, K, Ca, B, Zn, Mo, Na, CEC and lime requirement) being poorly estimated. Others including P, Mg, S, Cu, Fe and NH_3_ were estimated moderately well. This gives an indication of which soil parameters might be amenable to assessment using this approach, in terms of identifying nutrient deficiencies in soil that are “reflected” in crop spectral characteristics.

**Table 3:** Performance of neural network model estimating soil properties from crop spectra at Balruddery agricultural site. RMSE, Root Mean Squared Error.

Soil Property	Minimum	Maximum	Mean	R-Squared	RMSE
pH	5.5	6.4	6.01	0.15	0.18
K (ppm)	29	71	43.8	0.36	5.8
P (ppm)	103	459	235	0.21	55
Mg (ppm)	113	203	154	0.49	11
Ca (ppm)	1385	2142	1727	0.17	120
S (ppm)	5	17	9.17	0.40	1.72
Mn (ppm)	23	61	41.5	0.55	5.1
Cu (ppm)	7.1	14.5	9.89	0.41	1.35
B (ppm)	0.81	1.27	1	0.12	0.11
Zn (ppm)	2	10.7	4.55	0.18	1.3
Mo (ppm)	0.01	0.12	0.05	0.16	0.03
Fe (ppm)	509	981	762	0.44	71
Na (ppm)	21	80	31	0.11	10.6
CEC (meq/100 g)	11.8	16.2	13.6	0.20	0.77
Lime required (tons/ha)	3	9	5.25	0.09	1.9
NH_3_ (ppm)	1.9	122	8.03	0.46	14.4
NO_3_ (ppm)	10.8	117.3	45.8	0.71	10.4
Available N (kg/ha)	39	543	162	0.67	61

## 4. Discussion and Conclusions

We have demonstrated the ability of a low-cost and relatively technologically unsophisticated system to produce visible-range spectra from crops in field conditions. These spectra have been shown to be of sufficient quality to allow crop discrimination and the evaluation of soil nutrient conditions to a certain extent. The accuracy rates achieved (r^2^ values of 0.55 for Mn, 0.71 for NO_3_, 0.67 for Available N) are not earth-shatteringly good; however, they do demonstrate an ability to obtain soil nutrient information from crop spectra. Further work is required in improving the PHYLIS system design and ease of use, and in developing spectral libraries that allow crop and soil properties and characteristics to be investigated (particularly by capturing spectra directly from the soil and relating these to soil composition information).

For a wide range of crops, we have shown that variation of within-crop spectra, as captured with PHYLIS, is less than between-crop variation. We have also shown that within the preprocessing options explored here, the best preprocessing appears to be no preprocessing at all; this is an interesting and unexpected result, as we had anticipated that existing methods of highlighting important structure within the spectra would improve the system performance.

Another important observation relates to illumination intensity. Adjusting the amount of light entering the system is important for system performance, although the ability to discriminate between crops appears to be crop-specific. However, as it is difficult to know which crops will be harder to discriminate when the illumination levels are non-optimal, this indicates that when using a system such as PHYLIS, care should be taken to achieve illumination levels that avoid either overexposure or underexposure. Both of these situations result in a loss of discrimination ability and result in flat intensity curves. Therefore, it is important to achieve proper use of the illumination control on the system to produce a moderated light intensity for the camera capturing the spectral image.

Discrimination of crop types using PHYLIS-derived spectra indicates a certain level of potential. We achieved the ability to discriminate between broad crop types (beans, potatoes and grains) but were unable to narrow the specificity to allow discrimination between grain types. This indicates that the data from PHYLIS (and possibly, therefore, visible-range spectra in general) can allow broad crop or vegetation discrimination but not species- or variety-level discrimination. It also appears that data from PHYLIS cannot be used to discriminate between cropping strategies within individual crop types. The effects of different management options on soil and crop condition are strong and can be measured in a number of ways such as soil organic matter content and structure, or crop yield and nutrient composition; the fact that we have not successfully discriminated the management types in sensing of the crops implies that not all of these changes are visible to the sensor. There is, therefore, some likely use of a PHYLIS-like system in an agricultural context, but it is not unlimited in its potential. Future work will prioritise investigating the ability of the system to discriminate between a wider range of vegetation types, and also between species within broad land cover categories (e.g., types of broadleafed trees or grassland species).

For the estimation of soil properties, the story is the same, at least when using spectra from the crop: some properties can be estimated quite well, while others cannot. We do not yet know if this is true of spectra captured directly from the soil, but we suspect that it will be the case. Of particular interest is the demonstrable ability to use these spectra to evaluate soil nitrogen status and that of a number of other nutrients directly from observation of the crop. There are a number of potential applications to this, including the detection of nutrient deficiencies in agricultural systems and the application of this information for precision agriculture.

What we have demonstrated here is a prototype for rapid, in-field assessment of crop condition and nutrient status. Further work is required to fully realise the potential of this system, with the main barrier at the moment being the need to download the spectra from the system camera and process it separately. Future work will focus on (1) development of crop and soil spectral libraries; (2) improvements to the overall design of PHYLIS; (3) development of an integrated data capture-process-visualisation system to improve speed and performance; and (4) evaluation of the data from PHYLIS in comparison to data from a commercial spectrometer, using the same wavelength ranges.

One of the design changes that we will be exploring is the removal of the infrared filter from the camera that we have used, in order to explore whether it is possible to extend the wavelength range of the device. The infrared filter in digital cameras is intended to limit the wavelength range of light that falls on the camera sensor, which may be sensitive at wavelengths up to (or even beyond) 1000 nm. Removing the filter could potentially double the wavelength range of the system.

## Figures and Tables

**Table 1:** Confusion matrix for neural network model trained to discriminate four crop types.

	Beans	Potatoes	Barley	Wheat	User’s Accuracy	Total
Beans	20	1	2	1	0.83	24
Potatoes	1	22	2	1	0.85	26
Barley	0	0	11	11	0.50	22
Wheat	3	1	9	11	0.46	24
Producer’s Accuracy	0.83	0.92	0.46	0.46		
Total	24	24	24	24		

**Table 2:** Confusion matrix for neural network model trained to discriminate two cropping systems.

	Sustainable Cropping	Conventional Cropping	User’s Accuracy	Total
Sustainable cropping	30	20	0.60	50
Conventional cropping	18	28	0.61	46
Producer’s Accuracy	0.62	0.58		
Total	48	48		
